# Comparative efficacy and safety for different chemotherapy regimens used concurrently with thoracic radiation for locally advanced non-small cell lung cancer: a systematic review and network meta-analysis

**DOI:** 10.1186/s13014-019-1239-7

**Published:** 2019-03-29

**Authors:** Tingting Liu, Zheng He, Jun Dang, Guang Li

**Affiliations:** Department of Radiation Oncology, The First Hospital of China Medical University, 155 Nanjing Road, Heping District, Shenyang, 110001 China

**Keywords:** Locally advanced non-small cell lung cancer, Concurrent chemoradiation, Network meta-analysis

## Abstract

**Background:**

It remains unknown which is the most preferable regimen used concurrently with thoracic radiation for locally advanced non-small cell lung cancer (NSCLC). We performed a network meta-analysis to address this important issue.

**Methods:**

PubMed, Embase, Cochrane Library, Web of Science and major international scientific meetings were searched for relevant randomized controlled trials (RCTs). Overall survival (OS) data was the primary outcome of interest, and progression-free survival (PFS), and serious adverse events (SAEs) were the secondary outcomes of interests, reported as hazard ratio (HR) or odds ratio (OR) and 95% confidence intervals (CIs).

**Results:**

14 RCTs with a total of 2975 patients randomized to receive twelve categories of treatments were included in the meta-analysis. Direct comparison meta-analysis showed that etoposide-cisplatin (EP) was more effective than paclitaxel-cisplatin/carboplatin (PC) in terms of OS (HR = 0.85, 95% CI: 0.77–0.94) and PFS (HR = 0.66, 95% CI: 0.47–0.95). In network meta-analysis, all regimen comparisons did not produce statistically significant differences in survival. Based on treatment ranking of OS and the benefit-risk ratio, S-1-cisplatin (SP) was likely to be the most preferable regimen for its best efficacy and low risk of causing SAEs. Uracil/tegafur-cisplatin (UP) and pemetrexed-cisplatin/carboplatin (PP) were ranked the second and third respectively. Gemcitabine-cisplatin (GP) and PC + Cetuximab (PC-Cet) appeared to be the worst and second-worst regimens for their poor efficacy and poor tolerability.

**Conclusions:**

Based on efficacy and tolerability, SP is likely to be the most preferable regimen used concurrently with thoracic radiation for locally advanced NSCLC, followed by UP and PP. Further direct head-to-head studies are needed to confirm these findings.

**Electronic supplementary material:**

The online version of this article (10.1186/s13014-019-1239-7) contains supplementary material, which is available to authorized users.

## Introduction

Lung cancer remains the leading cause of cancer-associated deaths globally. Non-small cell lung cancer (NSCLC) accounts for approximately 85% of all lung cancer cases [1] and about 30% of NSCLC patients have locally advanced diseases [2]. For unresectable, locally advanced NSCLC patients with good performance status, concurrent chemoradiotherapy (CCRT) remains a standard of care. A meta-analysis of individual patient data from nine randomised trials [3] has shown that platin-based CCRT improved survival compared to RT alone. Currently, etoposide-cisplatin (EP) and paclitaxel-cisplatin/carboplatin (PC) are two most common concurrent chemotherapy regimens [4–7]. However, the outcomes have remained unsatisfactory. Recently, a number of new generation chemotherapy agents such as vinorelbine [8–11], docetaxel [12–14], gemcitabine [12], irinotecan [15], pemetrexed [9, 16, 17], and S-1 [10, 14] are increasingly being used concurrently with thoracic radiation for locally advanced NSCLC patients, and have shown good efficacy in clinical trials. However, direct comparison trials between these new options and conventional regimens like EP are still lacking, and therefore, there are still unresolved questions around which is the optimal chemotherapy regimen used concurrently with thoracic radiation.

Network meta-analysis is the best way to solve aforementioned questions, which enable indirect comparisons to account for missing head-to-head data and multiple regimen comparisons. The study aimed to perform a network meta-analysis to estimate the relative efficacy and tolerability of different agents based concurrent chemotherapy regimens, attempting to identify the most preferable regimen used concurrently with thoracic radiation for locally advanced NSCLC.

## Materials and methods

### Literature search strategy

This meta-analysis was conducted in accordance with the Preferred Reporting Items for Systematic Reviews and Meta-analysis (PRISMA) criteria [18]. PubMed, Embase, Cochrane Library, Web of Science, and major international scientific meetings were searched for the available studies published before October 31, 2018, using the strategy as shown in Additional file 1: Table S1. The reference lists of retrieved studies were manually scanned for relevant additional studies missed by the electronic search.

### Inclusion and exclusion criteria

Studies were included if they met the following criteria: (1) types of studies: randomized controlled trials (RCTs); (2) types of participants: participants with a histopathological diagnosis of locally advanced NSCLC; (3) types of interventions: one or more regimens for experimental arm, and the presence of a control for comparison; and (4) outcome: reported overall survival (OS) and/or progression-free survival (PFS) data. Studies were excluded if any of the following criteria were applied: (1) letters, editorials, case reports, and reviews; and (2) survival data could not be extracted from the literature.

### Data extraction

The data were extracted by two investigators independently. The following data were extracted from each study: first author, years of publication, duration of the study, country of origin, treatments, numbers of patients (experimental arm/control arm), data of OS, PFS, objective response rate (ORR), and serious adverse events (SAEs).

### Quality assessment

The methodological quality of RCTs was assessed by Cochrane risk of bias tool [19], which consists of the following five domains: sequence generation, allocation concealment, blinding, incomplete data, and selective reporting. A RCT was finally rated as “low risk of bias” (all key domains indicated as low risk), “high risk of bias” (one or more key domains indicated as high risk), and “unclear risk of bias”.

### Statistical analysis

The primary outcome was OS, and the secondary outcomes were PFS, ORR, and SAEs. Hazard ratios (HRs) or odds ratios (ORs) and their 95% confidence intervals (CIs) were used as summary statistics. For direct comparisons, standard pairwise meta-analysis (PWMA) was performed. A statistical test for heterogeneity was performed using the chi-square (*χ*^2^) and *I*-square (*I*^2^) tests with the significance set at *I*^2^ > 50% or *P* < 0.10.

The Bayesian network-meta analysis (NMA) was performed in a random-effect model using Markov chain Monte Carlo methods [20, 21] in JAGS and the GeMTC package in R (https://drugis.org/software/r-packages/gemtc). For each outcome measure, four independent Markov chains were simultaneously run for 20,000 burn-ins and 100,000 inference iterations per chain to obtain the posterior distribution. The traces plot and Brooks-Gelman-Rubin method were used to assess the convergence of model [22]. Treatment effects were estimated by HR/OR and corresponding 95% CI.

Network consistency was assessed with node-split models by statistically testing between direct and indirect estimates within treatment loop [23]. To rank probabilities of all available treatments, the surfaces under the cumulative ranking curve (SUCRAs) were calculated [24]. SUCRA equals one if the treatment is certain to be the best and zero if it’s certain to be the worst [24]. To jointly compare the efficacy and tolerability of each treatment and to assess their benefit-risk ratios, we ranked them based simultaneously on the SUCRA value of OS and tolerability (1-SUCRA_SAEs_) in the ranking plot. Lastly, comparison-adjusted funnel plot was used to detect the presence of small-study effects or publication bias [25].

## Results

### Literature search results and characteristics of included studies

**Fig. 1:**
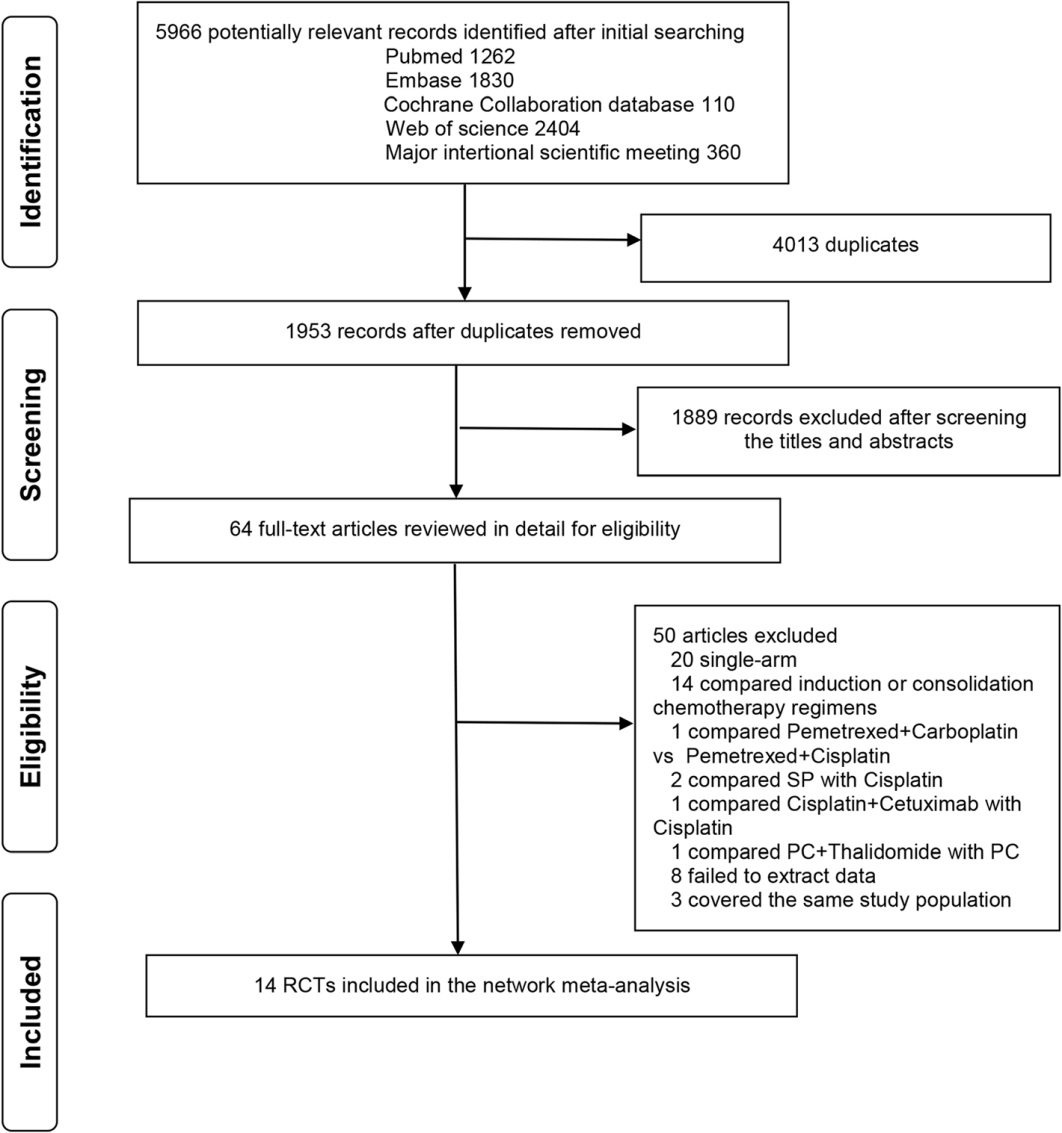
Literature search and selection. SP, S-1-cisplatin; PC, paclitaxel-cisplatin/carboplatin

The literature search results and study selection process are shown in Fig. 1. The initial search retrieved 5966 studies. After removing the duplicates, 1953 citations were identified, and 1889 of them were excluded through an abstract review. The remaining 64 studies were screened through a full-text review for further eligibility. Finally, 14 RCTs with 2975 patients randomized to receive the twelve categories of treatments were included in the meta-analysis. The twelve treatments were EP, PC, pemetrexed-cisplatin/carboplatin (PP), S-1-cisplatin (SP), uracil/tegafur (UFT)-cisplatin (UP), vinorelbine-cisplatin (NP), gemcitabine-cisplatin (GP), docetaxel-cisplatin (DP), irinotecan-carboplatin (IC), mitomycin-vindesine-cisplatin (MVP), PC + cetuximab (PC-Cet), and PP + cetuximab (PP-Cet), respectively. One trial comparing cisplatin-pemetrexed vs. carboplatin-pemetrexed [26] was excluded because the two regimens were regarded as the same category of treatment (PP) in this meta-analysis. Two trials comparing SP vs. cisplatin alone were excluded due to that cisplatin alone is not commonly used clinically. Of the 14 included trials, twelve were two-arm studies, and the rest two was three-arm studies [12, 15]. The study characteristics are shown in Table 1.

**Table 1:** Characteristics of included trials

Trial	Design	Time	Region	Primary	Treatment	Median follow-up	Sample	Median	Histology(%)	Consolidation	Radiotherapy	Radiotherapy
		Range		Endpoint		(months)	Size	Age	(SCC/non-SCC)	Chemotherapy	Dose(Gy)	Technology
Liang/2017 [4]	III	2007–2011	China	OS	EP	73	95	59	68.4/31.6	50.5%	60–66	3D
					PC		96	57	64.6/35.4	35.4%		
Wang/2012 [5]	II	2004–2007	China	OS	EP	46	33	55.4	69.7/30.3	75.8%	60–66	3D
					PC		32	60.9	62.5/37.5	59.4%		
Shuayb/2018 [6]	II	NR	Bangladesh	NR	EP	NR	30	NR	NR	NR	45	NR
					PC		30	NR	NR	NR		
Bradley/2015 [7]	III	2007–2011	USA	OS	PC-Cet	21	237	64	43/57	67%	60–74	3D
					PC	23	228	64	46/54	67%		
Sugawara/2013 [8]	II	2006–2009	Japan	ORR	UP	20	35	62	42.9/57.1	60%	60	2D − +3D
					NP		31	61	35.5/64.5	58.1%		
Zhao/2016 [9]	III	2008–2012	China	OS	NP	29	48	57.4	67/33	88%	60.6–74.4	3D
					PP		52	60.3	60/40	87%		
Sasaki T/2018 [10]	II	2009–2012	Japan	OS	SP	NR	54	60	31.5/68.5	59.3%	60	3D
					NP		54	62	31.5/68.5	44.4%		
Curran Jr./2011 [11]	III	1994–1998	USA	OS	NP	132	195	60	38/62	NR	60	NR
					EP		187	63	37/63	NR		
Oh/2013 [12]	III	2005–2007	Korea	ORR	PC	Over 36	33	64	72.7/27.3	63.6%	60–66	3D
					DP		29	61.5	69/31	65.5%		
					GP		31	64	64.5/35.5	64.5%		
Segawa/2010 [13]	III	2000–2005	Japan	OS	MVP	NR	101	NR	52.5/37.5	NR	60	2D
					DP		99	NR	44.4/55.6	NR		
Takiguchi/2018 [14]	II	2011–2014	Japan	OS	SP	48	53	NR	26.4/73.6	NR	60	NR
					DP		53	NR	20.8/79.2	NR		
Yamamoto/2010 [15]	III	2001–2005	Japan	OS	MVP	NR	146	63	47.9/52.1	41%	60	2D
					IC		147	62	42.2/57.8	29.3%		
					PC		147	63	48.3/51.7	49.7%		
Senan/2016 [16]	III	2008–2012	USA	OS	PP	22	301	59.5	100	76%	60–66	3D
					EP	23	297	58.7	100	74.3%		
Govindan/2011 [17]	II	2005–2008	USA	OS	PP	32	48	65	35/65	69.8%	70	3D
					PP-Cet		53	66	34/66	85.4%		

Abbreviations:*OS* overall survival, *ORR* overall response rate, *SP* S-1-cisplatin, *UP* UFT-cisplatin, *NP* vinorelbine-cisplatin, *EP* etoposide-cisplatin, *MVP* mitomycin-vindesine-cisplatin, *DP* docetaxel-cisplatin, *PC* paclitaxel-cisplatin/carboplatin, *PP* pemtrexed-cisplatin/carboplatin, *IC* irinotecan-carboplatin, *GP* gemcitabine-cisplatin, *Cet* cetuximab, *SCC* squamous cell carcinoma, *2D* two-dimensional radiotherapy, *3D* three-dimensional conformal radiotherapy, *NR* not reported

### Assessment of included trial

The risk of bias in included RCTs was summarized in Additional file 2: Figure S1. Seven trials [5, 6, 8, 12, 14, 15, 17] were judged to be unclear risk of bias, as they had more than three domains indicating as unclear risk. The remaining trials were rated with a low risk of bias. No trial was judged to be high risk of bias. Funnel plot analysis in term of OS did not indicate any evident risk of publication bias (Additional file 3: Figure S2).

### Conventional pairwise meta-analysis

Results of single trial and direct comparison meta-analysis are shown in Table 2. Direct comparison meta-analysis was feasible only for EP vs. PC. EP was more effective than PC in terms of OS (HR = 0.85, 95% CI: 0.77–0.94) and PFS (HR = 0.66, 95% CI: 0.47–0.95). No significant differences were observed in ORR and overall SAEs between the two arms. PC had a trend higher risk of causing grade≧3 radiation pneumonitis (RP) than EP (OR = 0.48, 95% CI: 0.21–1.1; *P* = 0.08).

**Table 2:** Results of single trial and direct comparison meta-analysis

Treatment	Study	OS	PFS	ORR	Overall SAEs	RP	Heterogeneity I^2^(%)				
		HR(95%CI)	HR(95% CI)	OR(95% CI)	OR(95% CI)	OR(95% CI)	OS	PFS	ORR	SAE	RP
EP vs PC	[4–6]	0.85 (0.77–0.94)	0.66 (0.47–0.95)	1.0 (0.86–1.2)	1.2 (0.81–1.4)	0.48 (0.21–1.1)	0	0	59	0	27
PC-Cet vs PC	[7]	1.1 (0.84–1.4)	0.99 (0.8–1.2)	NR	1.2 (1.1–1.4)	0.56 (0.27–1.2)					
UP vs NP	[8]	0.86 (0.35–2.1)	0.68 (0.35–1.3)	1.6 (0.53–5.1)	0.47 (0.29–0.76)	0.88 (0.12–6.6)					
NP vs PP	[9]	1.7 (0.81–3.4)	1.6 (0.91–2.6)	1.4 (0.38–5.4)	1.9 (1.1–3.3)	5.7 (0.26–120.6)					
SP vs NP	[10]	0.85 (0.49–1.5)	0.37 (0.15–0.94)	0.79 (0.31–2.0)	0.67 (0.50–0.88)	1.3 (0.32–5.0)					
NP vs EP	[11]	0.93 (0.75–1.1)	NR	1.3 (0.82–1.9)	0.91 (0.81–1.0)	0.86 (0.50–1.5)					
PC vs DP	[12]	1.0 (0.33–3.3)	1.1 (0.48–2.3)	0.67 (0.23–2.0)	0.67 (0.39–1.2)	4.7 (0.22–101.6)					
PC vs GP	[12]	0.77 (0.34–1.8)	0.67 (0.3–1.5)	1.1 (0.40–3.0)	0.80 (0.45–1.4)	1.9 (0.17–22.5)					
DP vs GP	[12]	0.65 (0.28–1.5)	0.72 (0.36–1.5)	1.7 (0.56–4.9)	1.2 (0.70–2.0)	0.34 (0.01–8.8)					
MVP vs DP	[13]	1.2 (0.8–1.7)	1.2 (0.89–1.7)	0.64 (0.33–1.2)	1.8 (1.5–2.0)	0.67 (0.24–1.8)					
SP vs DP	[14]	0.81 (0.39–1.7)	1.1 (0.63–1.9)	1.2 (0.52–2.8)	0.49 (0.35–0.68)	0.10 (0.31–3.2)					
MVP vs IC	[15]	0.98 (0.74–1.3)	0.89 (0.69–1.1)	1.5 (0.95–2.5)	1.8 (1.5–2.0)	0.33 (0.06–1.6)					
MVP vs PC	[15]	0.95 (0.72–1.3)	1.1 (0.82–1.4)	1.2 (0.71–1.9)	2.1 (1.8–2.4)	0.33 (0.06–1.6)					
IC vs PC	[15]	1.1 (0.79–1.4)	1.1 (0.86–1.5)	0.75 (0.47–1.2)	1.2 (1.0–1.4)	1.0 (0.31–3.2)					
PP vs EP	[16]	0.98 (0.79–1.2)	0.86 (0.71–1.0)	1.1 (0.81–1.6)	0.96 (0.85, 1.1)	0.68 (0.21–2.2)					
PP vs PP-Cet	[17]	1.1 (0.58–2.0)	1.1 (0.62–1.8)	1.3 (0.54–3.3)	0.99 (0.74–1.3)	1.1 (0.32–3.6)					

Abbreviations: *OS* overall survival, *PFS* progression-free survival, *ORR* objective response rate, *SAEs* serious adverse events, *RP* radiation pneumonitis, *HR* hazard ratio, *CI* confidence interval, *OR* odds ratio, *EP* etoposide-cisplatin, *PC* paclitaxel-cisplatin/carboplatin, *UP* uracil/tegafur(UFT)-cisplatin, *NP* vinorelbine-cisplatin, *PP* pemetrexed-cisplatin/carboplatin, *SP* S-1-cisplatin, *DP* docetaxel-cisplatin, *GP* gemcitabine-cisplatin, *MVP* mitomycin-vindesine-cisplatin, *IC* irinotecan-carboplatin, *Cet* cetuximab, *NR* not reported

### Network meta-analysis

**Fig. 2:**
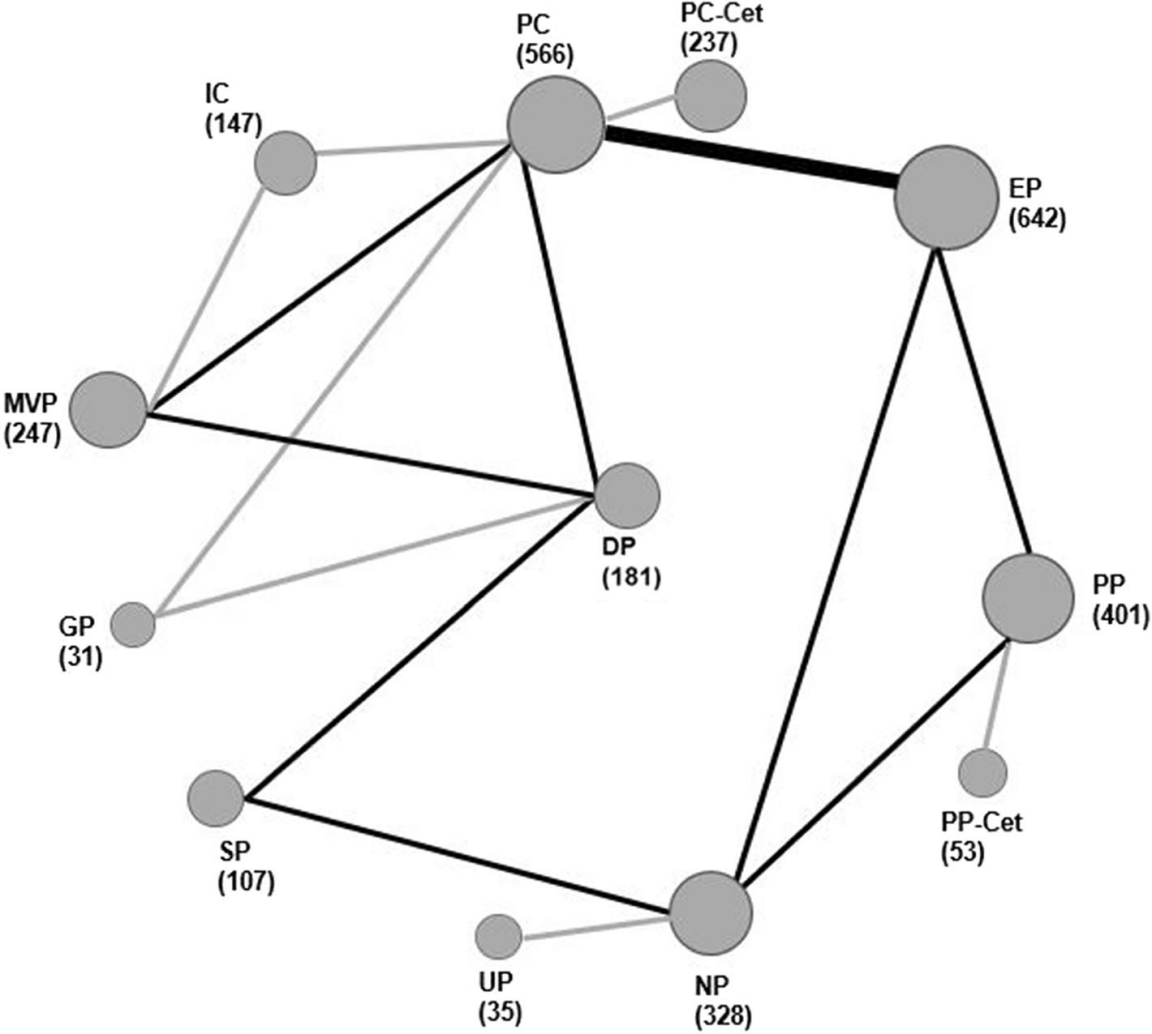
Network of eligible comparisons for the Bayesian network meta-analysis. The size of the nodes is proportional to the number of patients (in parentheses) randomized to receive the treatment. The width of the lines is proportional to the number of trials (beside the line) comparing the connected treatments. EP, etoposide-cisplatin; PC, paclitaxel-cisplatin/carboplatin; SP, S-1-cisplatin; UP, uracil/tegafur (UFT)-cisplatin; PP, pemtrexed-cisplatin/carboplatin; NP, vinorelbine-cisplatin; DP, docetaxel-cisplatin; IC, irinotecan-carboplatin; GP, gemcitabine-cisplatin; MVP, mitomycin-vindesine-cisplatin; Cet, cetuximab

The network plot established for NMA is shown in Fig. 2. Results of the NMA were presented in Additional file 4: Table S2. In term of OS, EP showed a trend significant advantage over PC (HR = 0.83, 95% CI: 0.65–1.0; *P* = 0.05). Other regimen comparisons did not produce statistically significant differences. With regard to PFS and ORR, no significant differences were observed for all regimen comparisons. As for overall SAEs and RP, MVP showed significantly higher risk of SAEs in comparison to each regimen except GP and PC-Cet. DP was more likely to cause SAEs than SP (OR = 0.51, 95% CI: 0.30–0.86) and UP (OR = 0.38, 95% CI: 0.15–0.96). NP resulted in a higher and a trend higher risk of SAEs than UP (OR = 0.47, 95% CI: 0.24–0.94) and SP (OR = 0.63, 95% CI: 0.38–1.0; *P* = 0.05), respectively. PC had a trend higher risk of grade≧3 RP than PP (OR = 0.053, 95% CI: 0.00064–1.0; *P* = 0.05) and EP (OR = 0.19, 95% CI: 0.016–1.1; *P* = 0.06).

### Inconsistency assessment and treatment ranking

There were three independent closed loops in the network for OS, ORR, overall SAEs: EP-NP-PP, PC-DP-MVP, and PC-DP-SP-NP-EP; two independent closed loops for PFS: PC-DP-MVP; and PC-DP-SP-NP-EP. Analysis of inconsistency showed that the NMA results were similar to the PWMA results for the four outcomes, which suggested the consistency between the direct and indirect evidence (Additional file 5: Figure S3).

The treatment rankings based on SUCRA are shown in Table 3. In terms of OS, SP was the most effective treatment (0.80), followed by PP (0.70), PP-Cet (0.68), UP (0.68), NP (0.65), EP (0.60), and DP (0.60). With regard to PFS, PP was the most effective treatment (0.89), followed by PP-Cet (0.86), EP (0.75), and UP (0.74). As for overall SAEs, UP was ranked as the least toxic regimen (0.03), followed by SP (0.11) and PP (0.33); MVP (1.0) was ranked as the highest toxic regimen. In term of RP, PC-Cet (0.83), PC (0.69) were ranked the highest and second-highest risk of causing grade≧3 RP respectively.

**Table 3:** SUCRA values for four outcomes

OS		PFS		SAEs		RP	
Treatment	SUCRA	Treatment	SUCRA	Treatment	SUCRA	Treatment	SUCRA
SP	0.80	PP	0.89	UP	0.03	MVP	0.32
PP	0.70	PP-Cet	0.86	SP	0.11	PP	0.36
PP-Cet	0.68	EP	0.75	PP	0.33	PP-Cet	0.37
UP	0.68	UP	0.74	PP-Cet	0.38	NP	0.41
NP	0.65	DP	0.55	PC	0.41	UP	0.42
EP	0.60	SP	0.48	NP	0.50	DP	0.46
DP	0.60	NP	0.41	EP	0.50	EP	0.47
MVP	0.35	PC-Cet	0.36	GP	0.60	GP	0.47
PC	0.29	PC	0.35	PC-Cet	0.69	SP	0.50
IC	0.28	MVP	0.28	IC	0.7	IC	0.69
PC-Cet	0.22	IC	0.17	DP	0.75	PC	0.69
GP	0.15	GP	0.15	MVP	1.0	PC-Cet	0.83

Abbreviations: *OS* overall survival, *PFS* progression-free survival, *SAE* serious adverse events, *RP* radiation pneumonitis, *SUCRA* surface under the cumulative ranking curve, *EP* etoposide-cisplatin, *PC* paclitaxel-cisplatin/carboplatin, *UP* uracil/tegafur(UFT)-cisplatin, *NP* vinorelbine-cisplatin, *PP* pemetrexed-cisplatin/carboplatin, *SP* S-1-cisplatin, *DP* docetaxel-cisplatin, *GP* gemcitabine-cisplatin, *MVP* mitomycin-vindesine-cisplatin, *IC* irinotecan-carboplatin, *Cet* cetuximab

**Fig. 3:**
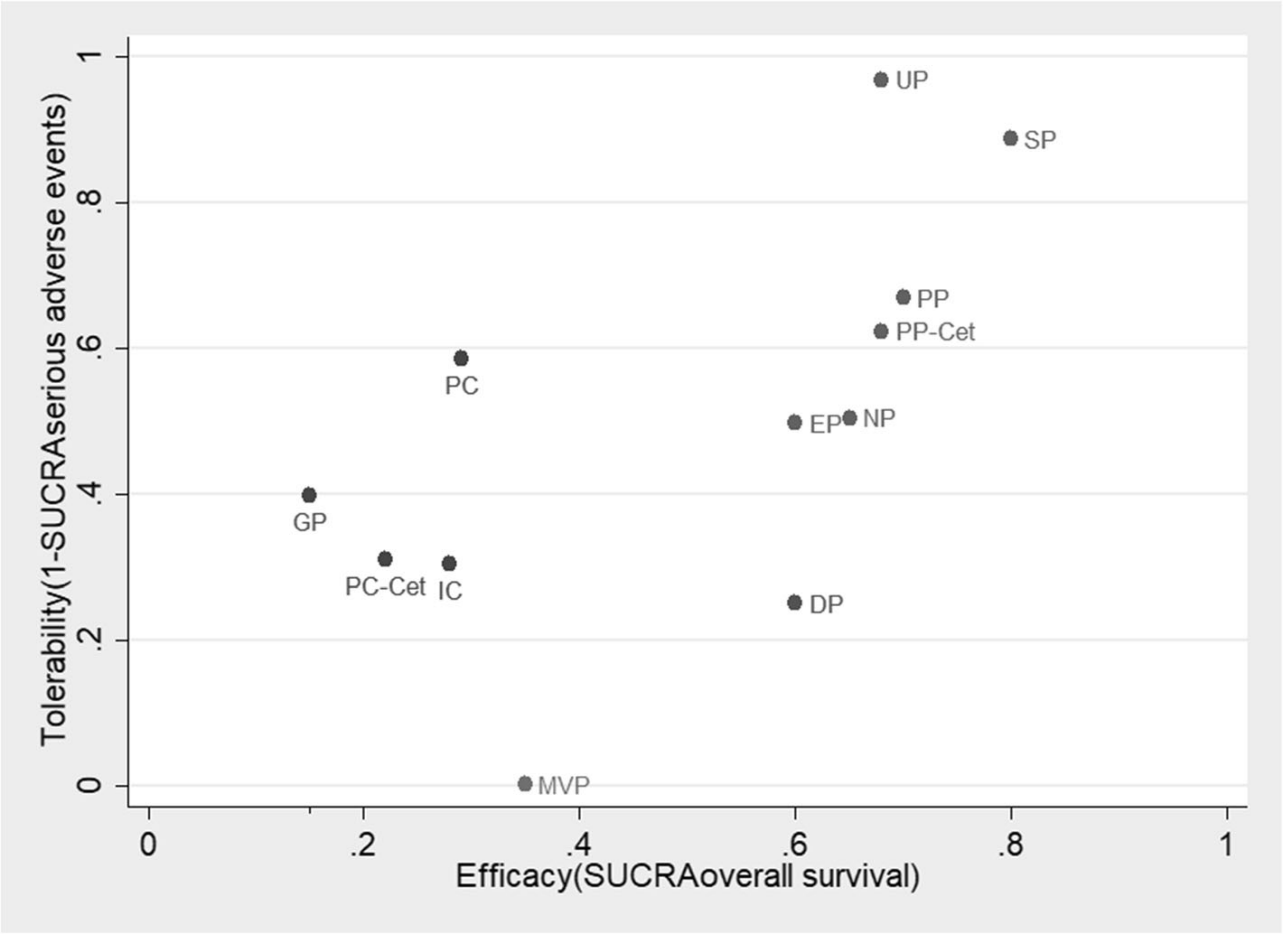
Ranking plot based simultaneously on efficacy (x-axis: SUCRA value of overall survival) and tolerability (y-axis: 1-SUCRA value of serious adverse events). SUCRA, surface under the cumulative ranking curves; SP, S-1-cisplatin; UP, uracil/tegafur (UFT)-cisplatin; PP, pemtrexed-cisplatin/carboplatin; EP, etoposide-cisplatin; PC, paclitaxel-cisplatin/carboplatin; NP, vinorelbine-cisplatin; DP, docetaxel-cisplatin; IC, irinotecan-carboplatin; GP, gemcitabine-cisplatin; MVP, mitomycin-vindesine-cisplatin; Cet, cetuximab

To further assess the benefit-risk ratios of the twelve treatments simultaneously, we ranked them based on the SUCRA values of OS and tolerability (1-SUCRA _SAE_) in the ranking plot (Fig. 3). SP was likely to be the optimal because it had the most efficacy with low risk of causing SAEs. UP, PP, and PP-Cet were ranked the second, third, and fourth respectively. EP and NP also had good efficacy with moderate risk of causing SAEs, and were ranked the fifth and sixth respectively. DP had similar efficacy to EP but with higher risk of causing SAEs. GP and PC-Cet appeared to be the worst and second worst for their poor efficacy and poor tolerability.

## Discussion

To our knowledge, this is the first network meta-analysis assessing the comparative efficacy and tolerability of all major chemotherapy regimens used concurrently with thoracic radiation for patients with locally advanced NSCLC. It showed that SP was likely to be the most preferable regimen based on the benefit-risk ratio. S-1 is a new oral fluoropyrimidine formulation that comprises tegafur, 5-chloro-2, 4-dihydroxypyridine, and potassium oxonate. Data from several single-arm phase II trials [27–29] have consistently shown that SP with concurrent thoracic radiation is a promising treatment for patients with locally advanced NSCLC, with OS rate of 51–70% at 2 years and 43–52.9% at 5 years, which appear to be superior to other concurrent chemotherapy regimens employed in other clinical trials. In a more recent randomized phase II trial, SP provided higher 2 years OS rate (75.6% vs. 68.5%) and longer PFS (14.8 months vs. 12.3 months) than NP [10]. Although there was no statistically significant difference, the PFS curve showed more favourable results for the SP arm over the long term, and tolerability was better. Similarly, SP resulted in a superior 2, 5-years OS with less toxicity compared with DP in another recent randomized phase II trial of SP or DP with concurrent thoracic radiotherapy for inoperable stage III NSCLC [14]. However, to date SP has not been compared directly with other established regimens. In our NMA, SP had the highest probability (80%) of being the most effective treatment in improving OS and with a low risk of causing SAEs, suggesting it to be a promising candidate as a standard regimen for locally advanced NSCLC. Besides, UFT (another oral fluoropyrimidine formulation) plus cisplatin (UP) also showed a good efficacy with better tolerability in the present NMA. Nevertheless, S-1 is not approved by the FDA. While data from 2 randomized phase II trials are promising regarding the use or S-1-cisplatin for CCRT in locally advanced NSCLC, a phase III trial is warranted before any recommendations regarding its use in this setting can be made.

PP has been the standard first-line treatment option in patients with metastatic non-squamous NSCLC. However, no clear survival advantages are reported for locally advanced case. A phase III trial (PROCLAIM) compared concurrent chemoradiation using PP vs. EP in stage III non-squamous NSCLC [16]. No statistically significant difference in OS was observed. Median PFS were 11.4 and 9.8 respectively, trended in favor of PP. However, PP had significantly lower Grade 3 or higher SAEs compared to EP (*P* = 0.01). In our NMA, PP was ranked third-best regimen in term of the benefit-risk ratio. The addition of Cetuximab did not demonstrate a survival advantage compared with PP alone. It should be noted that all included patients (except PROCLAIM trial) were with mixed histological types. In a phase III trial comparing PP and GP in patients with advanced NSCLC, PP resulted in a superior OS in non-squamous patients but did a worse survival for patients with squamous histology tumors compared with GP [30]. Further head-to-head comparisons, for locally advanced disease, with other concurrent regimens according to histological type are also needed.

EP and PC, two most common regimens administered currently with radiation to date, have recently been compared directly in several clinic trials. In a most recent multicenter randomized phase III trial [4] comparing EP and PC with concurrent thoracic radiotherapy in unresectable stage III NSCLC, the 3-year OS was significantly higher in the EP arm than in the PC arm. Similar survival advantage in EP arm was also found in two phase II trials [5, 6]. In our NMA, EP failed to show survival advantage compared with any other regimens though with PC. Based on the benefit-risk ratio, EP showed better efficacy but with moderate risk of causing SAEs, and were ranked sixth-best after SP, UP, PP, and NP.

RP remains one of the most common side effects for patients treated with chemotherapy concurrently with thoracic radiation, and different chemotherapy regimens have been reported to be related to the differences in RP risk [5, 31]. In a phase II trial of concurrent EP or PC and thoracic radiotherapy for stage III NSCLC [5], the rate of grade ≥ 2 RP was 25% in the PE arm and 48.5% in the PC arm (*P* = 0.09). Data from a large meta-analysis of predictors of RP showed that concurrent PC resulted in five times the risk of grade ≥ 2 RP compared with EP [31]. In our NMA, PP and PC were ranked the lowest and highest risk of causing grade ≥ 3 RP, respectively. Other regimens such as NP, UP, DP, EP, GP, and SP, were with similarly moderate risk of causing grade ≥ 3 RP.

Molecularly targeted agents and immunotherapy have a defined role for metastatic NSCLC. The efficacy of these new classes of agents for locally advanced disease is undergoing investigation. In the 2017 Annual Meeting of the American Society of Clinical Oncology, Yu et al. reported a multicenter phase II trial of erlotinib vs. EP with concurrent thoracic radiotherapy for stage III NSCLC with epidermal growth factor receptor activating mutation [32]. Comparing with EP, median PFS of erlotinib arm was significantly improved (27.86 vs. 6.41 months, *P* < 0.001). However, the sample size is relatively small (21 in EP arm and 20 in erlotinib arm). These findings need a large sample size of phase III study to confirm. Morever, a recent phase III trial (PACIFIC) [33] compared PD-L1 inhibitor durvalumab with placebo in patients with stage III unresectable NSCLC, not progressing after chemoradiotherapy. The main chemotherapy regimens used for CCRT were cisplatin or carboplatin based regimens; in addition, 25.8% of the patients in the durvalumab group and 28.7% of those in the placebo group had received induction chemotherapy before definitive chemoradiotherapy. Patients receiving durvalumab had a three-fold increase in median PFS, and with a reduction in the risk of progression of 48%. Although the PACIFIC trial has demonstrated the obvious advantage of consolidation durvalumab after chemoradiation, identification of patients who really benefit from the addition of durvalumab and the optimal concurrent chemotherapy regimen in combine with consolidation immune checkpoint inhibitor are warranted.

This network meta-analysis has a number of limitations. Firstly, in common with other meta-analyses, data were collected and analyzed basis of results reported from trials, instead of individual patient data. Secondly, all studies except PROCLAIM trial included patients with mixed histological types. Thus, we could not assess survival differences between regimens according to histological types. Thirdly, different toxicity criteria were used, and radiotherapy features (such as counturing, planning and delivery) were inconsistent in individual RCTs. Moreover, chemotherapy regimens used in different countries were studied togheter in the meta-analysis. These might lead to heterogeneity and inconsistency. Finally, some HRs of OS or PFS were calculated from the Kaplan–Meier curve due to that they were not directly reported in the articles. This may result in bias.

## Conclusions

Based on efficacy and tolerability, SP is likely to be the most preferable regimen used concurrently with thoracic radiation for locally advanced NSCLC, followed by UP and PP. GP and PC-Cet appeared to be the worst and second-worst regimens for this population. Further direct head-to-head, well-designed, prospective studies are needed to confirm these findings.

## Additional files

Additional file 1: **Table S1.** Search strategy (DOC 62 kb)

Additional file 2: **Figure S1.** Assessment of risk of bias. A: Methodological quality graph: authors’ judgment about each methodological quality item presented as percentages across all included studies; B: Methodological quality summary: authors’ judgment about each methodological quality item for each included study, “+” low risk of bias; “?” unclear risk of bias; “-” high risk of bias. (JPEG 17793 kb)

Additional file 3: **Figure S2.** Comparison-adjusted funnel plots of publication bias test for overall survival. SP, S-1-cisplatin; UP, uracil/tegafur (UFT)-cisplatin; PP, pemtrexed-cisplatin/carboplatin; EP, etoposide-cisplatin; PC, paclitaxel-cisplatin/carboplatin; NP, vinorelbine-cisplatin; DP, docetaxel-cisplatin; IC, irinotecan-carboplatin; MVP, mitomycin-vindesine-cisplatin; GP, gemcitabine-cisplatin; Cet, cetuximab. (JPG 6940 kb)

Additional file 4: **Table S2.** Results of network meta-analysis (DOC 117 kb)

Additional file 5: **Figure S3.** Inconsistency evaluation by node-splitting analyses. (a) overall survival; (b) progression-free survival; (c) objective response rate; (d) serious adverse events. EP, etoposide-cisplatin; PC, paclitaxel-cisplatin/carboplatin; PP, pemtrexed-cisplatin/carboplatin; MVP, mitomycin-vindesine-cisplatin; SP, S-1-cisplatin; NP, vinorelbine-cisplatin; DP, docetaxel-cisplatin. (JPG 9280 kb)

## Abbreviations

CCRT: Concurrent chemoradiotherapy
CI: Confidence interval
DP: Docetaxel-cisplatin
EP: Etoposide-cisplatin
GP: Gemcitabine-cisplatin
HR: Hazard ratio
IC: Irinotecan-carboplatin
MVP: Mitomycin-vindesine-cisplatin
NMA: Network-meta analysis
NP: Vinorelbine-cisplatin
NSCLC: Non-small cell lung cancer
OR: Odds ratio
ORR: Objective response rate
OS: Overall survival
PC: Paclitaxel-cisplatin/carboplatin
PC-Cet: PC + cetuximab
PFS: Progression-free survival
PP: Pemetrexed-cisplatin/carboplatin
PP-Cet: PP + cetuximab
PWMA: Pairwise meta-analysis
RCT: Randomized controlled trial
RP: Radiation pneumonitis
SAE: Serious adverse event
SP: S-1-cisplatin
SUCRA: Surfaces under the cumulative ranking curve
UFT: Uracil/tegafur
UP: Uracil/tegafur-cisplatin
