# Supplementary material for: Comparative efficacy and safety for different chemotherapy regimens used concurrently with thoracic radiation for locally advanced non-small cell lung cancer: a systematic review and network meta-analysis
Source: Radiat Oncol. 2019 Mar 29;14:55. doi: 10.1186/s13014-019-1239-7 (PMC6441209; doi:10.1186/s13014-019-1239-7)
Supplement: Supplementary file 1 — Table S1. Search strategy (DOC 62 kb) [file 13014_2019_1239_MOESM1_ESM.doc]

**Table S1** Search strategy

**a:** Search strategy in PubMed

| # | Query |
| --- | --- |
| #1 | “Lung Neoplasms”[mh] |
| #2 | Lung Neoplasms[tiab] OR Neoplasms, Lung[tiab] OR Lung Neoplasm[tiab] OR Neoplasm, Lung[tiab] OR Neoplasms, Pulmonary[tiab] OR Neoplasm, Pulmonary[tiab] OR Pulmonary Neoplasm[tiab] OR Pulmonary Neoplasms[tiab] OR Lung Cancer[tiab] OR Cancer, Lung[tiab] OR Cancers, Lung[tiab] OR Lung Cancers[tiab] OR Pulmonary Cancer[tiab] OR Cancer, Pulmonary[tiab] OR Cancers, Pulmonary[tiab] OR Pulmonary Cancers[tiab] OR Cancer of the Lung[tiab] OR Cancer of Lung[tiab] |
| #3 | "Carcinoma, Non-Small-Cell Lung"[mh] |
| #4 | Carcinoma, Non Small Cell Lung[tiab] OR Carcinomas, Non-Small-Cell Lung[tiab] OR Lung Carcinoma, Non-Small-Cell[tiab] OR Lung Carcinomas, Non-Small-Cell[tiab] OR Non-Small-Cell Lung Carcinomas[tiab] OR Nonsmall Cell Lung Cancer[tiab] OR Non-Small-Cell Lung Carcinoma[tiab] OR Non Small Cell Lung Carcinoma[tiab] OR Carcinoma, Non-Small Cell Lung[tiab] OR Non-Small Cell Lung Cancer[tiab] OR NSCLC[tiab] |
| #5 | #1 OR #2 OR #3 OR #4 |
| #6 | "Chemoradiotherapy"[mh] |
| #7 | Chemoradiotherapies[tiab] OR Radiochemotherapy[tiab] OR Radiochemotherapies[tiab] OR Concurrent Chemoradiotherapy[tiab] OR Chemoradiotherapies, Concurrent[tiab] OR Chemoradiotherapy, Concurrent[tiab] OR Concurrent Chemoradiotherapies[tiab] OR Synchronous Chemoradiotherapy[tiab] OR Chemoradiotherapies, Synchronous[tiab] OR Chemoradiotherapy, Synchronous[tiab] OR Synchronous Chemoradiotherapies[tiab] OR Concurrent Radiochemotherapy[tiab] OR Radiochemotherapies, Concurrent[tiab] OR Radiochemotherapy, Concurrent Concomitant Chemoradiotherapy[tiab] OR Chemoradiotherapies, Concomitant[tiab] OR Chemoradiotherapy, Concomitant[tiab] OR Concomitant Chemoradiotherapies[tiab] OR Concomitant Radiochemotherapy[tiab] OR Concomitant Radiochemotherapies[tiab] OR Radiochemotherapies, Concomitant[tiab] OR Radiochemotherapy, Concomitant[tiab] OR CCRT |
| #8 | #6 OR #7 |
| #9 | Randomized Controlled Tial[pt] |
| #10 | Controlled Cinical Trial[pt] |
| #11 | Randomized[tiab] |
| #12 | Placebo[tiab] |
| #13 | Randomly[tiab] |
| #14  #15 | Trial[tiab]  Drug Therapy[sh] |
| #16 | Groups[tiab] |
| #17 | #9 OR #10 OR #11 OR #12 OR #13 OR #14 OR #15 OR #16 |
| #18 | Animals[mh] |
| #19 | Humans[mh] |
| #20 | #18 NOT #19 |
| #21 | #17 NOT #20 |
| #22 | #5 AND #8 AND #21 |

**b:** Search strategy in Embase

| # | Query |
| --- | --- |
| #1 | ‘lung cancer’/exp |
| #2 | ‘non small cell lung cancer’/exp |
| #3 | 'non small cell':ab,ti |
| #4 | ‘nsclc’:ti,ab |
| #5 | #1 OR #2 OR #3 OR #4 |
| #6 | ‘chemoradiotherapy'/exp |
| #7 | 'chemoradi*':ab,ti OR 'radiochemo*':ab,ti OR 'ccrt':ab,ti |
| #8 | #6 OR #7 |
| #9 | #5 AND #8 |
| #10 | 'trial':ab,ti |
| #11 | 'random*':ab,ti |
| #12 | 'clinical trial'/de OR 'controlled clinical trial'/de OR 'randomized controlled trial'/de |
| #13 | #10 OR #11 OR #12 |
| #14 | #9 AND #13 |

**c:** Search strategy in Cochrane Library

| # | Query |
| --- | --- |
| #1 | MeSH descriptor: [Carcinoma, Non-Small-Cell Lung] explode all trees |
| #2 | MeSH descriptor: [Lung Neoplasms] explode all trees |
| #3 | ((lung OR pulmon*) AND (neoplas* OR cancer OR carcinoma* OR tumour* or tumor*)) |
| #4 | non-small cell* |
| #5 | non small cell* |
| #6 | nonsmall cell* |
| #7 | Nsclc |
| #8 | #1 OR #2 OR #3 OR #4 OR #5 OR #6 OR #7 |
| #9 | MeSH descriptor: [Chemoradiotherapy] explode all trees |
| #10 | chemoradi* |
| #11 | radiochemo* |
| #12 | CCRT |
| #13 | #9 OR #10 OR #11 OR #12 |
| #14 | #8 AND #13 |

**d:** Search strategy in Web of Science

| # | Query |
| --- | --- |
| #1 | TS=("lung cancer" OR "non-small cell lung cancer" OR NSCLC OR ((lung OR pulmon*) AND (neoplas* OR cancer OR carcinoma* OR tumour* or tumor*))) |
| #2 | TS=("chemoradi*" OR "radiochemo*" OR "ccrt" OR "chemoradiotherapy") |
| #3 | TS=("randomized controlled trial" OR "controlled clinical trial" OR "clinical trial" OR "random*" OR "rct*" OR "crossover" OR "masked” OR “blind*" OR "placebo*") |
| #4 | #1 AND #2 AND #3 |
