# Supplementary material for: Comparative efficacy and safety for different chemotherapy regimens used concurrently with thoracic radiation for locally advanced non-small cell lung cancer: a systematic review and network meta-analysis
Source: Radiat Oncol. 2019 Mar 29;14:55. doi: 10.1186/s13014-019-1239-7 (PMC6441209; doi:10.1186/s13014-019-1239-7)
Supplement: Supplementary file 4 — Table S2. Results of network meta-analysis (DOC 117 kb) [file 13014_2019_1239_MOESM4_ESM.doc]

**Table S2.** Results of network meta-analysis

| a. Hazard ratios(HR) with 95%CI for overall survival(OS) | | | | | | | | | | | |
| --- | --- | --- | --- | --- | --- | --- | --- | --- | --- | --- | --- |
| SP |  |  |  |  |  |  |  |  |  |  |  |
| 0.92(0.49-1.9） | PP |  |  |  |  |  |  |  |  |  |  |
| 0.99(0.39-2.7) | 1.1(0.54-2.1) | PP-Cet |  |  |  |  |  |  |  |  |  |
| 0.97(0.34-2.9) | 1.1(0.37-2.9) | 0.98(0.28-3.3) | UP |  |  |  |  |  |  |  |  |
| 0.84(0.48-1.5) | 0.92(0.55-1.3) | 0.85(0.36-1.9) | 0.87(0.34-2.2) | NP |  |  |  |  |  |  |  |
| 0.84(0.47-1.5) | 0.92(0.61-1.3) | 0.85(0.39-1.8) | 0.87(0.32-2.3) | 0.99(0.73-1.4) | EP |  |  |  |  |  |  |
| 0.83(0.45-1.5） | 0.90(0.45-1.6） | 0.84(0.31-2.1) | 0.85(0.28-2.5) | 0.98(0.56-1.7) | 0.99(0.57-1.6) | DP |  |  |  |  |  |
| 0.72(0.38-1.4) | 0.79(0.42-1.3) | 0.73(0.28-1.7) | 0.74(0.26-2.1) | 0.85(0.52-1.4) | 0.86(0.54-1.3) | 0.87(0.56-1.4) | MVP |  |  |  |  |
| 0.70(0.38-1.3) | 0.77(0.46-1.1) | 0.71(0.30-1.5) | 0.72(0.26-2.0) | 0.82(0.57-1.2) | **0.83(0.65-1.0)** | 0.84(0.52-1.4) | 0.97(0.66-1.4) | PC |  |  |  |
| 0.71(0.36-1.4) | 0.78(0.40-1.3) | 0.72(0.28-1.7) | 0.73(0.25-2.2) | 0.84(0.49-1.5) | 0.85(0.52-1.3) | 0.86(0.49-1.5) | 0.99(0.66-1.5) | 1.0(0.68-1.5) | IC |  |  |
| 0.65(0.32-1.3) | 0.71(0.37-1.2) | 0.66(0.26-1.5) | 0.67(0.23-1.9) | 0.77(0.45-1.4) | 0.78(0.47-1.2) | 0.78(0.42-1.5) | 0.90(0.52-1.6) | 0.93(0.62-1.4) | 0.91(0.51-1.6) | PC-Cet |  |
| 0.58(0.25-1.4) | 0.63(0.27-1.3) | 0.58(0.20-1.6) | 0.59(0.18-2.0) | 0.68(0.32-1.5) | 0.69(0.33-1.4) | 0.70(0.35-1.4) | 0.80(0.39-1.6) | 0.83(0.42-1.6) | 0.81(0.38-1.7) | 0.89(0.42-2.0) | GP |
| b. Hazard ratios(HR) with 95%CI for progression-free survival (PFS) | | | | | | | | | | | |
| SP |  |  |  |  |  |  |  |  |  |  |  |
| 1.5(0.69-3.6) | PP |  |  |  |  |  |  |  |  |  |  |
| 1.6(0.52-5.0) | 1.0(0.48-2.3) | PP-Cet |  |  |  |  |  |  |  |  |  |
| 1.4(0.48-4.1) | 0.91(0.30-2.7) | 0.87(0.22-3.3) | UP |  |  |  |  |  |  |  |  |
| 0.95(0.50-1.9) | 0.62(0.31-1.2) | 0.60(0.21-1.7) | 0.68(0.28-1.6) | NP |  |  |  |  |  |  |  |
| 1.3(0.61-3.2) | 0.87(0.49-1.6) | 0.83(0.33-2.2) | 0.96(0.30-3.1) | 1.4(0.65-3.2) | EP |  |  |  |  |  |  |
| 1.0(0.52-2.1） | 0.68(0.29-1.5) | 0.65(0.21-2.0) | 0.74(0.22-2.4) | 1.1(0.47-2.4) | 0.77(0.36-1.5) | DP |  |  |  |  |  |
| 0.84(0.37-1.9) | 0.55(0.23-1.2) | 0.52(0.16-1.6) | 0.60(0.17-2.0) | 0.88(0.35-2.1) | 0.63(0.30-1.2) | 0.81(0.46-1.4) | MVP |  |  |  |  |
| 0.89(0.41-2.0) | 0.58(0.29-1.2) | 0.56(0.19-1.6) | 0.64(0.19-2.1) | 0.94(0.41-2.1) | 0.67(0.41-1.0) | 0.86(0.48-1.6) | 1.1(0.62-1.9) | PC |  |  |  |
| 0.75(0.29-1.9) | 0.49(0.19-1.2) | 0.47(0.14-1.5) | 0.54(0.15-1.9) | 0.79(0.30-2.1) | 0.56(0.25-1.1) | 0.73(0.34-1.6) | 0.90(0.48-1.7) | 0.84(0.45-1.5) | IC |  |  |
| 0.90(0.33-2.5) | 0.59(0.23-1.5) | 0.56(0.16-1.8) | 0.65(0.17-2.4) | 0.95(0.33-2.7) | 0.68(0.30-1.4) | 0.87(0.37-2.1) | 1.1(0.47-2.5) | 1.0(0.53-1.9) | 1.2(0.50-2.9) | PC-Cet |  |
| 0.63(0.22-1.8) | 0.41(0.14-1.1) | 0.39(0.10-1.4) | 0.45(0.11-1.8) | 0.66(0.22-2.0) | 0.47(0.17-1.2) | 0.60(0.26-1.4) | 0.75(0.30-1.9) | 0.70(0.30-1.6) | 0.83(0.31-2.3) | 0.69(0.24-2.0) | GP |
| c. Odds ratios (OR) with 95% CI for objective response rate (ORR) | | | | | | | | | | | |
| SP |  |  |  |  |  |  |  |  |  |  |  |
| 1.3(0.37-5.0) | PP |  |  |  |  |  |  |  |  |  |  |
| 1.7(0.30-11.0) | 1.3(0.39-4.4) | PP-Cet |  |  |  |  |  |  |  |  |  |
| 0.61(0.11-3.5) | 0.47(0.08-2.5) | 0.36(0.04-2.8) | UP |  |  |  |  |  |  |  |  |
| 1.0(0.36-2.8) | 0.79(0.28-1.9) | 0.58(0.12-2.6) | 1.7(0.40-6.8) | NP |  |  |  |  |  |  |  |
| 1.5(0.49-4.6) | 1.1(0.50-2.5） | 0.83(0.19-3.6) | 2.3(0.49-12.0) | 1.4(0.69-3.2) | EP |  |  |  |  |  |  |
| 0.98(0.36-2.6） | 0.75(0.20-2.5) | 0.57(0.10-3.1) | 1.6(0.27-9.7) | 0.96(0.32-2.9) | 0.68(0.0.23-1.8) | DP |  |  |  |  |  |
| 1.4(0.40-4.4) | 1.0(0.28-3.5) | 0.78(0.13-4.3) | 2.2(0.36-1.4) | 1.3(0.41-4.2) | 0.94(0.32-2.5) | 1.4(0.59-3.2) | MVP |  |  |  |  |
| 1.4(0.44-4.2) | 1.1(0.36-2.8) | 0.82(0.16-3.8) | 2.3(0.41-13.0) | 1.4(0.53-3.5) | 0.98(0.47-1.8) | 1.5(0.59-3.4) | 1.0(0.45-2.3) | PC |  |  |  |
| 2.0(0.50-7.6) | 1.5(0.37-5.5) | 1.1(0.18-6.7) | 3.2(0.48-21.0) | 2.0(0.54-7.1) | 1.4(0.42-3.9) | 2.0(0.64-6.2) | 1.5(0.58-3.7) | 1.4(0.57-3.6) | IC |  |  |
| 1.6(0.35-7.0) | 1.2(0.25-5.5) | 0.93(0.12-6.5) | 2.6(0.34-20.0) | 1.6(0.36-6.8) | 1.1(0.27-4.1) | 1.1(0.34-3.8) | 1.2(0.31-4.5) | 1.1(0.34-3.8) | 0.81(0.19-3.5) | GP |  |
| d. Odds ratios (OR) with 95%CI for serious adverse events (SAEs) | | | | | | | | | | | |
| SP |  |  |  |  |  |  |  |  |  |  |  |
| 0.75(0.43-1.7) | PP |  |  |  |  |  |  |  |  |  |  |
| 0.74(0.34-2.1) | 0.99(0.54-1.8) | PP-Cet |  |  |  |  |  |  |  |  |  |
| 1.3(0.56-3.2) | 1.8(0.66-3.8） | 1.8(0.55-4.7) | UP |  |  |  |  |  |  |  |  |
| **0.63(0.38-1.0)** | 0.85(0.43-1.3) | 0.86(0.33-1.7) | **0.47(0.24-0.94)** | NP |  |  |  |  |  |  |  |
| 0.64(0.38-1.2) | 0.86(0.49-1.2) | 0.86(0.37-1.7) | 0.47(0.22-1.1) | 1.0(0.69-1.6) | EP |  |  |  |  |  |  |
| **0.51(0.30-0.86）** | 0.68(0.30-1.2) | 0.69(0.24-1.5) | **0.38(0.15-0.96)** | 0.81(0.45-1.5) | 0.80(0.44-1.3) | DP |  |  |  |  |  |
| **0.31(0.16-0.59)** | **0.41(0.18-0.74)** | **0.41(0.14-0.93)** | **0.23(0.09-0.60)** | **0.48(0.26-0.94)** | **0.48(0.26-0.81)** | **0.59(0.38-0.98)** | MVP |  |  |  |  |
| 0.67(0.39-1.3) | 0.90(0.46-1.5) | 0.91(0.36-1.9) | 0.50(0.22-1.3) | 1.1(0.65-1.9) | 1.1(0.72-1.5) | 1.3(0.86-2.2) | **2.2(1.5-3.6)** | PC |  |  |  |
| 0.55(0.27-1.2) | 0.73(0.30-1.4) | 0.74(0.25-1.8) | 0.40(0.15-1.2) | 0.86(0.44-1.9) | 0.86(0.44-1.6) | 1.1(0.59-2.1) | **1.8(1.1-3.1)** | 0.81(0.47-1.4) | IC |  |  |
| 0.54(0.25-1.3) | 0.73(0.30-1.5) | 0.74(0.25-1.8) | 0.40(0.15-1.2) | 0.85(0.43-2.0) | 0.85(0.44-1.6） | 1.1(0.54-2.3) | 1.8(0.91-3.8) | 0.81(0.47-1.4) | 0.99(0.47-2.2) | PC-Cet |  |
| 0.58(0.26-1.3) | 0.77(0.29-1.7) | 0.77(0.24-2.0) | 0.43(0.15-1.3) | 0.91(0.41-2.1) | 0.90(0.41-1.9） | 1.1(0.57-2.2) | 1.9(0.89-4.0) | 0.86(0.42-1.7) | 1.1(0.45-2.4) | 1.1(0.43-2.5) | GP |
| e. Odds ratios (OR) with 95%CI for radiation pneumonitis (RP) | | | | | | | | | | | |
| SP |  |  |  |  |  |  |  |  |  |  |  |
| 1.3(0.018-120) | PP |  |  |  |  |  |  |  |  |  |  |
| 1.4(0.0065-400) | 1.1(0.037-30.0) | PP-Cet |  |  |  |  |  |  |  |  |  |
| 0.52(0.003-54.0) | 0.39(0.0015-42.0) | 0.37(0.00055-110) | UP |  |  |  |  |  |  |  |  |
| 0.47(0.015-7.6) | 0.36(0.0068-6.9) | 0.33(0.0016-24.0) | 0.87(0.019-40.0) | NP |  |  |  |  |  |  |  |
| 0.39(0.0066-12.0) | 0.29(0.0097-3.7) | 0.26(0.002-15.0) | 0.73(0.0069-74.0) | 0.81(0.057-12.0) | EP |  |  |  |  |  |  |
| 0.16(0.0014-4.6） | 0.12(0.0007-6.1) | 0.11(0.000022-17.0) | 0.29(0.0011-53.0) | 0.34(0.0055-14.0) | 0.41(0.01-12.0) | DP |  |  |  |  |  |
| 0.25(0.0016-9.9) | 0.18(0.001-9.1) | 0.17(0.00032-25.0) | 0.45(0.0015-93.0) | 0.53(0.0074-23.0) | 0.64(0.015-17.0) | 1.6(0.09-24.0) | MVP |  |  |  |  |
| 0.073(0.00068-1.9) | **0.053(0.00064-1.0)** | 0.051(0.00017-3.4) | 0.13(0.00067-15.0) | 0.16(0.0042-2.7) | **0.19(0.016-1.1**) | 0.48(0.015-8.8) | 1.0(0.041-25.0) | PC |  |  |  |
| 0.073(0.00028-4.6) | 0.053(0.00021-3.1) | 0.051(0.000067-8.3) | 0.13(0.00029-34.0) | 0.16(0.0014-8.6) | 0.19(0.003-5.7) | 0.47(0.0073-19.0) | 0.30(0.0094-7.6) | 0.97(0.04-25.0) | IC |  |  |
| 0.041(0.00012-3.2) | 0.03(0.00011-1.8) | 0.029(0.000036-4.9) | 0.074(0.00014-19.0) | 0.09(0.00064-5.4) | 0.11(0.0015-3.3) | 0.27(0.0021-18.0) | 0.17(0.0018-10.0) | 0.55(0.022-13.0) | 0.56(0.006-48.0) | PC-Cet |  |
| 0.086(0.0002-14.0) | 0.063(0.00014-9.9) | 0.061(0.000047-22.0) | 0.17(0.00021-72.0) | 0.19(0.00087-27.0) | 0.23(0.0017-21.0) | 0.59(0.0044-56.0) | 0.36(0.0028-45.0) | 1.2(0.021-93.0) | 1.2(0.0081-230) | 2.3(0.012-430) | GP |

Abbreviations: EP, etoposide-cisplatin; PC, paclitaxel-cisplatin/carboplatin; UP, uracil/tegafur(UFT)-cisplatin; NP, vinorelbine-cisplatin; PP, pemetrexed-cisplatin/carboplatin; SP, S-1-cisplatin; DP, docetaxel-cisplatin; GP, gemcitabine-cisplatin; MVP, mitomycin-vindesine-cisplatin; IC, irinotecan-carboplatin; Cet, cetuximab.
